# Role of C-Terminal Domain and Membrane Potential in the Mobility of Kv1.3 Channels in Immune Synapse Forming T Cells

**DOI:** 10.3390/ijms23063313

**Published:** 2022-03-18

**Authors:** Veronika Sebestyén, Éva Nagy, Gábor Mocsár, Julianna Volkó, Orsolya Szilágyi, Ádám Kenesei, György Panyi, Katalin Tóth, Péter Hajdu, György Vámosi

**Affiliations:** 1 Department of Biophysics and Cell Biology, Faculty of Medicine, University of Debrecen, H-4032 Debrecen, Hungary; sebestyen.veronika@med.unideb.hu (V.S.); nagy.eva@med.unideb.hu (É.N.); mocsgab@med.unideb.hu (G.M.); volko.julianna@med.unideb.hu (J.V.); csoszi85@gmail.com (O.S.); kenesei.adam@med.unideb.hu (Á.K.); panyi@med.unideb.hu (G.P.); dr.toth.katalin@med.unideb.hu (K.T.); 2 Division Biophysics of Macromolecules, German Cancer Research Center, D-69120 Heidelberg, Germany; 3 Department of Biophysics and Cell Biology, Faculty of Dentistry, University of Debrecen, H-4032 Debrecen, Hungary

**Keywords:** immunological synapse, T cell, Kv1.3 channel, membrane depolarization, mobility, fluorescence correlation spectroscopy, live cell

## Abstract

Voltage-gated Kv1.3 potassium channels are essential for maintaining negative membrane potential during T-cell activation. They interact with membrane-associated guanylate kinases (MAGUK-s) via their C-terminus and with TCR/CD3, leading to enrichment at the immunological synapse (IS). Molecular interactions and mobility may impact each other and the function of these proteins. We aimed to identify molecular determinants of Kv1.3 mobility, applying fluorescence correlation spectroscopy on human Jurkat T-cells expressing WT, C-terminally truncated (ΔC), and non-conducting mutants of mGFP-Kv1.3. ΔC cannot interact with MAGUK-s and is not enriched at the IS, whereas cells expressing the non-conducting mutant are depolarized. Here, we found that in standalone cells, mobility of ΔC increased relative to the WT, likely due to abrogation of interactions, whereas mobility of the non-conducting mutant decreased, similar to our previous observations on other membrane proteins in depolarized cells. At the IS formed with Raji B-cells, mobility of WT and non-conducting channels, unlike ΔC, was lower than outside the IS. The Kv1.3 variants possessing an intact C-terminus had lower mobility in standalone cells than in IS-engaged cells. This may be related to the observed segregation of F-actin into a ring-like structure at the periphery of the IS, leaving much of the cell almost void of F-actin. Upon depolarizing treatment, mobility of WT and ΔC channels decreased both in standalone and IS-engaged cells, contrary to non-conducting channels, which themselves caused depolarization. Our results support that Kv1.3 is enriched at the IS via its C-terminal region regardless of conductivity, and that depolarization decreases channel mobility.

## 1. Introduction

Kv1.3 is a Shaker-type voltage-gated potassium channel consisting of four identical subunits, each containing six transmembrane α-helices. The voltage sensor of the channel is made up of the S4 α-helices containing positively charged amino acid residues in every third position [1]. Kv1.3 plays a significant role in T cell proliferation and T cell mediated immune responses. Together with the calcium-activated potassium channel KCa3.1, it is responsible for maintaining the negative membrane potential needed for calcium influx after the T cell had been activated via the T cell receptor (TCR) by its MHC-bound antigen ligand [2]. Because of their role in T cell responses, Kv1.3 channels are therapeutic targets in autoimmune disorders. It was described that although Kv1.3 inhibitors did not block immune synapse (IS) formation, they could reduce the activity of Ca-induced pathways in effector memory T cells of rheumatoid arthritis, type I diabetes, and multiple sclerosis patients, offering an opportunity to ameliorate autoimmune responses [3,4,5].

In addition to membrane potential regulation, Kv1.3 channels also participate in a number of protein–protein interactions, which may modulate the activity of various signaling pathways and of the channel itself [6,7]. We and other labs have shown that Kv1.3 co-localizes with the TCR/CD3 complex in lipid rafts of Jurkat T cells and redistributes to the IS formed between a cytotoxic T cell and a target B cell [6,8,9]. Accumulation and activity of ion channels at the IS may create a local ion milieu in the restricted volume of the synaptic cleft, which could modulate the function of voltage-gated channels and other molecules. We reported Kv1.3- and Ca^2+^-dependent periodic membrane potential oscillations in IS-engaged T cells, which could be important in T cell activation, IS formation, and molecular trafficking between the antigen presenting cell and the T cell [10]. Kv1.3 has also been reported to be in the molecular proximity of IS-stabilizer integrin β1 in T cells [11]. p56^lck^ and PKC kinases are intracellular elements of the T cell activation complex. Kv1.3 was shown to interact with these kinases through its accessory subunits (Kvβ) and hDlg adapter proteins [12,13,14,15,16]. Thus, Kv1.3 channels may be part of a signaling complex at the IS consisting of TCR/CD3, protein kinases, and integrin β1 [11,17]. Redistribution of Kv1.3 into the IS affects its function: its inactivation kinetics is accelerated probably due to modulation of its phosphorylation state, whereas its activation kinetics is slowed down likely because of partitioning into specific membrane domains [18,19].

Which molecular interactions lead to enrichment and arrest of Kv1.3 channels at the IS is still an unresolved issue. This behavior may be caused by the perturbation of trafficking, a lack of functionally important intracellular associations, or a change of diffusional mobility. Candidates for mediating protein–protein interactions are the C-terminal postsynaptic density zone (PDZ)-binding and SH3-binding domains, to which the PDZ-domains of intracellular scaffolding proteins such as membrane associated guanylate kinases (MAGUK-s like PSD-95 or SAP97) or actin nucleation proteins with SH3 domain (like HS1, cortactin, WASP, WAVE2) can be coupled, respectively [20,21]. PSD-95 was shown to regulate trafficking of the channel into the IS [22,23,24], while SAP-97 has no influence on Kv1.3 IS-redistribution in Jurkat cells [25]. Furthermore, cortactin is able to immobilize Kv1.3 channels upon actin polymerization in HEK cells, and HS1 and Kv1.3 colocalize and interact at the IS in Jurkat T cells [26].

Previously, we used fluorescence correlation spectroscopy (FCS) to assess the molecular mobility of interleukin-2 and -15 receptor subunits and MHC glycoproteins in T cells and found that these proteins slowed down in depolarized and moved faster in hyperpolarized membranes [27]. Molecular interactions and membrane potential changes may affect the function and mobility of Kv1.3 and of its interaction partners and, conversely, mobility may influence the stability of interactions, thereby the function of these protein complexes. Here, we asked how conductance and lack of the PDZ-binding domain-containing C-terminal, membrane depolarization, and IS formation influence the mobility of Kv1.3 channels.

## 2. Results

First, we carried out FCS experiments in the membrane of standalone Jurkat T cells expressing different variants of Kv1.3 channels to investigate the effect of the lack of C-terminal PDZ binding domain (ΔC mutant) and the effect of impaired conductivity (W384F mutant, denoted NON-CON) on channel mobility [25,28]. Autocorrelation curves were fitted to a model with two diffusion components (see Figure S1 for representative curves), a slow one in the range of 0.05–0.25 µm^2^/s representing diffusion of Kv1.3 in the cell membrane and a fast one (20–40 µm^2^/s) attributed to intracellular Kv1.3 (e.g., in vesicles). To confirm this, the mobility of Kv1.3 variants was also measured in the cytoplasm, which did not differ significantly from the fast component in the membrane (Figure S2). Previously, we obtained similar values for small oligomers (2–4) of EGFP in the cytoplasm [29].

**Figure 1:**
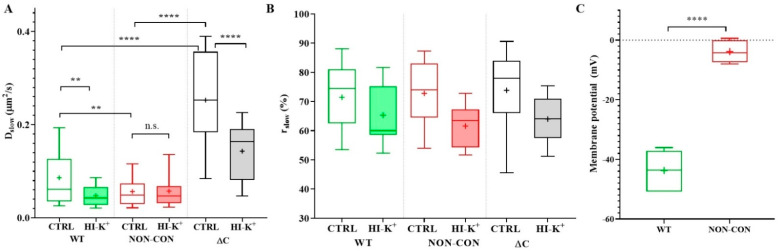
Diffusion properties of WT and mutant Kv1.3 channels in standalone cells. (**A**) Diffusion coefficient of the slow component. The mobility of the wild type mGFP-tagged Kv1.3 channel (WT) was compared to that of non-conducting point mutant (NON-CON) and C-terminal deleted mutant (ΔC) Kv1.3 channels (empty boxes) expressed in Jurkat cells. The NON-CON mutant channel has the lowest mobility, and the ΔC mutant has the highest mobility. Depolarization by high external K^+^ concentration using high K^+^ solution decreased the mobility of the WT and ΔC channels significantly (filled boxes). (**B**) Average proportions (*r_slow_*) of the slow component of the different Kv1.3 channels did not differ significantly. (**C**) Membrane potential of Jurkat cells expressing WT mGFP-Kv1.3 and the NON-CON mutant measured by patch clamp. On the box-and-whiskers plots, boxes represent the values of the 25th to 75th percentiles, whereas whiskers represent the 10th and 90th percentiles, the midline is the median value, and “+” marks the arithmetic mean. Mean values were compared with ANOVA and Student’s *t*-test. ** *p* < 0.01, **** *p* < 0.0001, n.s. not significant.

The slow diffusion coefficient of the ΔC channel was significantly greater than that of the WT or NON-CON channels (WT: 0.086 ± 0.061 μm^2^/s (mean ± SD), ΔC: 0.25 ± 0.11 μm^2^/s, *p* < 0.0001) (Figure 1A empty bars, see also Table 1). In contrast, the mobility of the NON-CON mutant relative to the WT channel was significantly lower (NON-CON: 0.056 ± 0.037 μm^2^/s, *p* < 0.01). The ratio of the slow component, *r_slow_*, did not change significantly in the different samples (Figure 1B). It is important to note that the WT channel sustains a negative membrane potential (app. −44 mV), whereas the membrane of cells expressing the NON-CON mutant channel is depolarized (−4 mV, *p* < 0.0001, Figure 1C).

**Figure 2:**
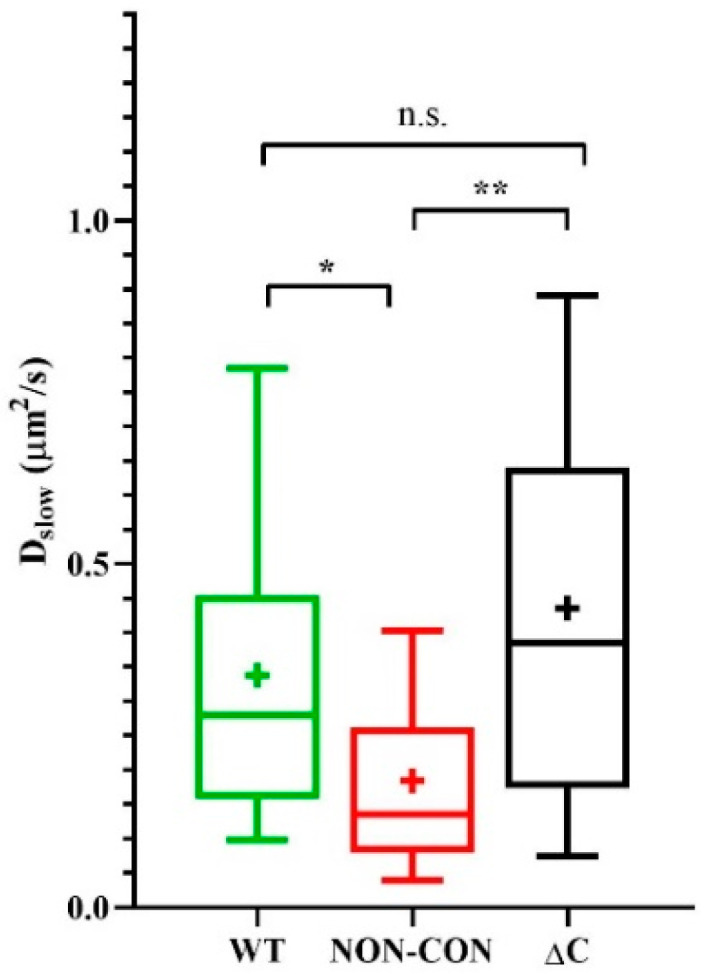
Mobility of MHC I heavy chain. Mobility of the MHC I glycoprotein was measured in standard solution in standalone Jurkat cells expressing WT, non-conductive point mutant (NON-CON), or C-terminal deleted mutant (ΔC) Kv1.3 channels. MHC I was labeled with Alexa546-W6/32 Fab. * *p* < 0.05, ** *p* < 0.01, n.s. not significant.

We reported previously that the mobility of MHC glycoproteins and IL-2/IL-15 receptors in lipid rafts of FT7.10 T lymphoma cells decreased upon depolarization by high-K^+^ solution or by the specific Kv1.3 channel blocker margatoxin (MgTx) [27]. Therefore, to dissect possible mechanisms affecting mobility of membrane proteins, we also evaluated the mobility of Alexa 546-W6/32 Fab-tagged MHC I molecules on the Jurkat lines expressing the three types of Kv1.3 channels. The slow diffusion coefficient of MHC I decreased significantly in cells expressing the NON-CON channels as compared to cells expressing WT or ΔC channels (NON-CON: 0.18 ± 0.13 μm^2^/s vs. WT: 0.34 ± 0.23 μm^2^/s (*p* < 0.05) and ΔC: 0.44 ± 0.31 μm^2^/s *p* < 0.01) (Figure 2). The decrease of mobility in the presence of the NON-CON mutant is in line with our previous observation, i.e., depolarization produced in different ways (high-K^+^ solution, margatoxin, non-conducting Kv1.3 channel) reduces MHC I mobility to a similar extent [27].

We also determined the mobility of the DiIC_18_ fluorescent lipid analogue, which is enriched in lipid rafts. The mobility of DiIC_18_ molecules did not differ significantly in the three cell lines (Figure S3, Table 1). The insensitivity to depolarization of the mobility of DiIC_18_ is in accordance with our previous data in T cells [27]. The unchanged mobility of MHC I and DiIC_18_ in ΔC channel-expressing cells suggests that deletion of the C-terminus influences the mobility of Kv1.3 specifically and does not cause generic changes in the membrane.

To study the effect of depolarizing conditions, we either bathed the cells in high-K^+^ buffer or added margatoxin (MgTx) to the standard extracellular solution. High-K^+^ solution depolarizes the membrane via several K^+^ channels and transporters, whereas MgTx is a specific inhibitor of Kv1.3 channels (K_d_ ≈ 50 pM) [30].

The mobility of WT and ΔC channels decreased in the high-K^+^ buffer, while that of the NON-CON channel did not alter (Figure 1A, filled columns). In the latter case, the high extracellular K^+^ concentration likely did not affect the membrane potential significantly because of the absence of the channel’s K^+^ conductance.

**Figure 3:**
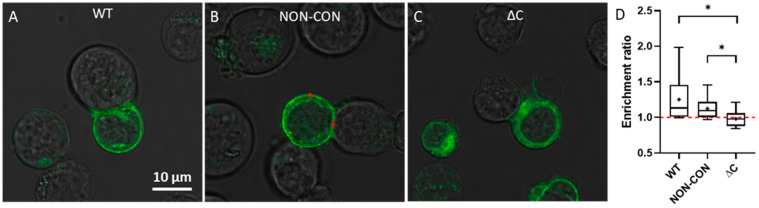
Transmission and fluorescence microscopic images of immunological synapses formed between a Raji B and Jurkat T cells expressing different mGFP-Kv1.3 variants. The red marks illustrate points inside and outside the IS where FCS measurements were typically carried out. Different versions of Kv1.3 are displayed: WT (**A**), NON-CON (**B**), and ΔC mutant (**C**). Panel (**D**) shows the extent of Kv1.3 enrichment at the IS according to Equation (3). The ratio of the average pixel intensity inside the IS and in the whole cell membrane was calculated from confocal images by using MATLAB (for details see Materials and Methods). * *p* < 0.05.

Next, we determined the distribution of Kv1.3 channels in T cells forming immunological synapses with SEE superantigen-treated Raji B cells. SEE contains two distinct binding sites for MHC II and one on the T cell receptor, thereby crosslinking these proteins [31]. Previously we used this model system to study Kv1.3 enrichment at the IS and IL-15 trans-presentation [25,32]. Here, we found that WT and NON-CON Kv1.3 variants were dominantly localized in the plasma membrane (Figure 3A,B), whereas the ΔC mutant exhibited a higher intracellular fraction (Figure 3C). We calculated the average pixel intensity ratio of the channels at the IS relative to the whole plasma membrane and found that the WT and NON-CON variants were enriched at the IS while the ΔC mutant was not (Figure 3D).

**Figure 4:**
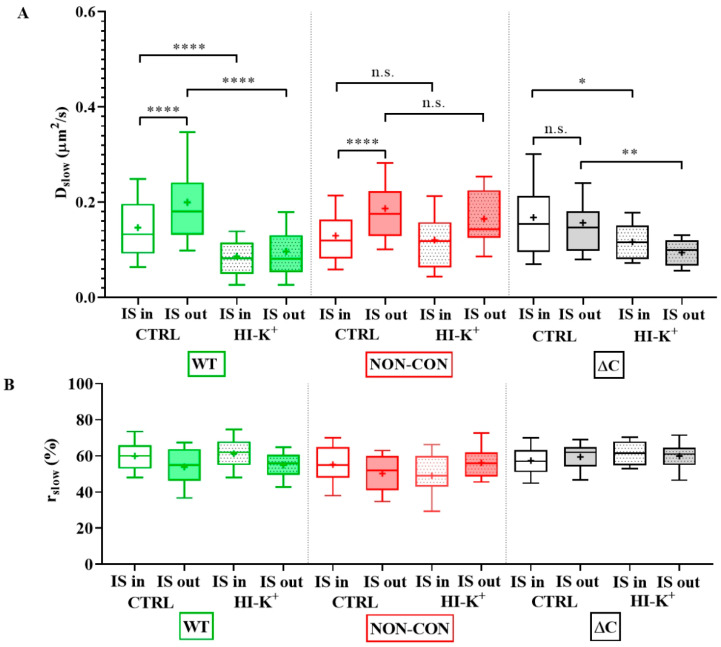
Mobility of wild type and mutant Kv1.3 channels on Jurkat T cells forming immunological synapses with Raji B cells at resting membrane potential and upon depolarization. (**A**) Slow diffusion coefficients of the WT and NON-CON channels decreased significantly inside the IS. However, that of the C-terminally truncated (ΔC) mutant did not change significantly. We also studied the effect of a depolarizing milieu. The mobility of WT and ΔC channels decreased significantly upon depolarization by high-K^+^ solution both inside and outside the IS. In contrast, the mobility of the NON-CON channels did not show significant alterations. (**B**) Average proportion of the slow diffusing component (*r_slow_*) was not significantly affected by being partitioned in the IS, by mutation, or by depolarization. * *p* < 0.05, ** *p* < 0.01, **** *p* < 0.0001, n.s. not significant.

As a next step, we studied the mobility of Kv1.3 variants in different regions of the membrane of IS-engaged cells. We found that mobility of the WT channel was significantly lower in the IS compared to that outside the IS (Figure 4A green symbols, and Table 1). We observed an even larger decrease in the mobility of WT mCherry-Kv1.3 channels at the IS in a murine model system of D10 T cells and CH12.LX B cells (Figure S4, Table 1). The mobility of the NON-CON mutant channel also decreased in the IS similar to the WT (*p* < 0.0001, Table 1). In contrast, the mobility of the ΔC mutant did not show a significant difference between IS- and non-IS-localized channels. Interestingly, the mobility of WT and NON-CON mutant Kv1.3 channels outside and even inside the IS was higher than in standalone cells (Table 1). On the contrary, mobility of ΔC channels was lower both inside and outside the IS than in standalone cells. It is important to note that whereas in standalone cells there was a stark difference between the mobility of WT, NON-CON, and ΔC channels, mobilities of all species were rather similar in IS-engaged cells both inside and outside the IS. The fraction of the slow diffusion component did not differ inside and outside the IS (Figure 4B), but it was ~10% less than in standalone cells (Figure 1B).

**Figure 5:**
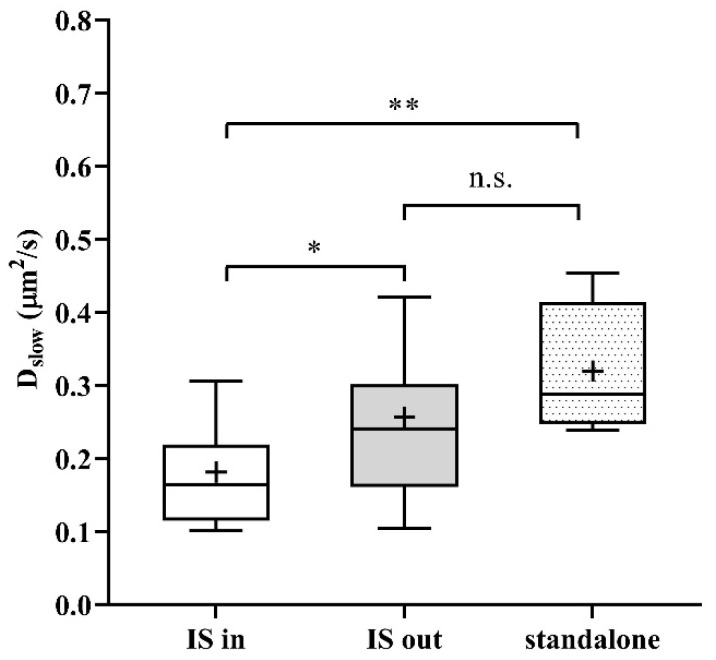
Mobility of IL-2 receptor α subunits labeled with Alexa 546-anti-Tac Fab on IS-forming Jurkat cells expressing WT mGFP-Kv1.3. IL-2Rα diffuses significantly more slowly in the IS than outside it, which may be the consequence of a more crowded environment. * *p* < 0.05, ** *p* < 0.01.

As a further control, we measured the mobility of the IL-2Rα receptor subunit in standalone and IS-engaged Jurkat cells expressing WT mGFP-Kv1.3. IL-2 receptors can also be found at the IS and are enriched in lipid raft domains [33,34]. The mobility of IL-2Rα decreased in the IS as compared to that outside the IS (Figure 5). Similar to ΔC channels, IL-2Rα was more mobile in standalone cells as compared to IS-engaged cells, contrary to Kv1.3 possessing an intact C-terminus.

To test the effect of depolarization on the mobility of Kv1.3 in the IS and outside the IS, we incubated IS forming T cell–B cell conjugates in high-K^+^ solution. Depolarization significantly decreased the mobility of WT and ΔC channels both inside and outside the IS but did not affect that of the NON-CON mutant significantly (Figure 4A).

Depolarization by specific Kv1.3 blocker MgTx (Figure S5) induced similar changes both on standalone and on IS-engaged cells as depolarization by high-K^+^ solution. For ΔC channels, depolarization by high-K^+^ or by MgTx almost completely canceled the difference in mobility between IS-engaged and standalone cells.

**Figure 6:**
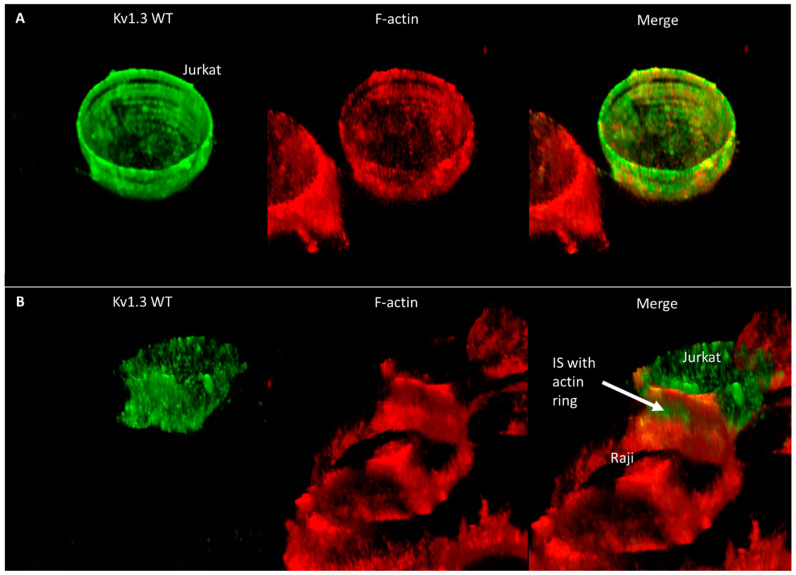
Distribution of Kv1.3 WT and F-actin in standalone and IS-engaged Jurkat cells. Kv1.3 was tagged with mGFP and F-actin was labeled with Alexa546-phalloidin. 3D reconstructions from z-stacks of confocal images are shown. (**A**) Standalone cells, (**B**) Jurkat cell in IS with Raji cell. The arrow marks the IS and the F-actin ring formed at its periphery.

To elucidate why the mobility of Kv1.3 is faster even at the IS than in standalone cells, we studied the distribution of the F-actin as a potential candidate that may influence the diffusion properties of membrane proteins. In standalone Jurkat cells, F-actin forms a more or less homogenous membrane skeleton under the membrane (Figure 6A and Figure S6A, Supplementary Video S1). On the other hand, in IS-forming Jurkat cells, actin creates a ring-like structure at the periphery of the contact region, and the center of the contact region is free of membrane-proximal actin (Figure 6B and Figure S6B, Supplementary Video S2); in the rest of the T cell, F-actin is polarized to the side opposite to the IS, leaving the in-between submembrane region void of F-actin, which may explain the higher mobility of the channels there. Further images and videos showing the co-distribution of Kv1.3 and F-actin in standalone and IS-forming Jurkat cells expressing the WT, NON-CON, and ΔC mutants are displayed in Figure S7 and Supplementary Videos S3–S8. According to these images, the F-actin distribution was similar in all cells, irrespective of the channel type expressed.

## 3. Discussion

The Kv1.3 potassium channel is one of the most important ion channels in maintaining the negative membrane potential during T cell activation. Therefore, inhibition of Kv1.3 activity is a promising strategy to treat various autoimmune diseases. Kv1.3 is also known to participate in several protein–protein interactions. Mobility may report on these molecular interactions in live cells; therefore, we studied how their C terminus, K^+^ conductivity, and redistribution to the IS influence their mobility in the membrane of a T cell line.

In standalone cells, the mobility of WT Kv1.3 was significantly lower than that of the ΔC mutant. In contrast, in cells expressing the WT and ΔC channels, the mobility of MHC I hardly differed from each other, so deletion of the C-terminus only had an effect on the channel itself. The mobility of DiIC_18_ did not change significantly in the cells expressing the three variants of the channels; thus, no general membrane rearrangement occurred that would alter membrane fluidity, supporting the specificity of the effect for Kv1.3 channels. Mobility of IL-2Rα in cells expressing WT Kv1.3 was 4-fold and 1.4-fold larger than that of the WT and ΔC channels. The difference is partly due to the size of the molecules: IL-2Rα contains a single transmembrane helix, whereas each of the 4 subunits of Kv1.3 contain 6 transmembrane (TM) helices. It is also known that PSD-95, a protein of the MAGUK family possessing a PDZ domain, guides the channel into a caveolin-containing, higher viscosity lipid domain [35,36]. This specific lipid environment may contribute to the lower mobility of the WT channel relative to the ΔC mutant, which lacks the PDZ-binding domain. Clustering plays an important role in protein mobility: earlier we have shown that silencing MHC I, a highly expressed member of IL-2R/IL-15R/MHC superclusters, leads to increased mobility of all the components of the cluster in T cells [37]. The low mobility of channels possessing the intact C terminus could be the consequence of intracellular interactions with scaffolding MAGUK proteins and cytoskeletal components. Interactions of the SH3 domain of the C-terminus [26] may also lower the mobility of the WT channel.

Next, we measured the effect of membrane depolarization. This question is relevant because membrane depolarization occurs due to K^+^ efflux from necrotic cells in inflamed tissues or in the tumor microenvironment where T cells often fulfill their functions. In tumor cells, depolarization can significantly affect migration, proliferation, and differentiation [38]. Functional coupling was demonstrated between Kv1.3 and integrin β1 in T cells: blocking the channel prevented migration of T cells [39]. The conductivity of Kv1.3 was found to be essential in other cells (platelet, melanoma) to facilitate migration and adherence via β1 integrin [11]. The mutation resulting in the loss of channel conductivity was shown by patch clamp to depolarize Jurkat cells, similar to HEK cells [40]. In standalone cells, the WT and the ΔC mutant were slowed down both in high-K^+^ solution and upon MgTx treatment, whereas the non-conducting mutant, which itself causes depolarization, already had low mobility even in standard solution, which was not further reduced by the above treatments. The mobility of MHC I decreased in NON-CON mutant-expressing cells compared to those expressing WT channels. Earlier, we found that depolarization by MgTx and high-K^+^ also decreased the mobility of MHC I, MHC II, and IL-2Rα, forming superclusters in lipid rafts of T lymphoma cells [27] but not of transferrin receptors partitioned into coated pits. On the other hand, the mobility of DiIC_18_ did not change significantly with high-K^+^ or MgTx treatment [27], and according to our present results, neither in cells expressing the NON-CON mutant. It is an intriguing question why several lipid-raft localized TM proteins slow down upon depolarization. TM proteins have positively charged amino acids in the cytoplasmic flanks of their TM regions (positive-inside, negative-not-inside rules) [41,42], as necessitated by the electrostatic potential of the cell membrane. These charges provide these proteins with a permanent dipole moment, due to which membrane potential changes may modulate their conformation and thereby their mobility and function [27].

For the first time, to our knowledge, we studied the mobility of Kv1.3 channels at IS’s formed between a T cell and an antigen presenting cell and compared it to other membrane proteins and to standalone cells. The mobility of the ΔC mutant in the IS was slightly lower than in standalone cells, and that of IL-2Rα was reduced 2-fold in the IS, which at first sight could be attributed to a higher degree of molecular crowding in the IS. In contrast, the mobility of WT channels was increased in IS-engaged cells both in the IS (2.5-fold) and outside the IS (1.8-fold), and this was true even for the NON-CON mutant, which assumed similar values as the WT. As discussed above, the low mobility in standalone cells is characteristic of channels possessing their intact C-terminus. Presumably, in standalone cells, the C-terminus interacts with some entity that significantly reduces mobility, but this interaction is not present in IS-engaged cells, even outside the IS. Clarifying the nature of this interaction requires further investigation. A candidate for this interacting partner is the actin cytoskeleton.

Whereas in standalone cells F-actin forms a more or less homogenous membrane skeleton below the membrane (Supplementary Videos S1 and S3, Figure 6A and Figure S6A), in IS-forming cells it is redistributed. It creates a ring-like structure at the periphery of the contact region, while the inside of the contact region is free of membrane-proximal F-actin (Supplementary Videos S2 and S4, Figure 6B and Figure S6B); in the rest of the T cell, F-actin is polarized to the side opposite to the IS, leaving some of the area in between free from F-actin. Similar observations on the ring-like distribution of F-actin were made in a T cell forming contact with CD3-coated coverslips [43].

Cortactin, an actin nucleation promoting factor, is able to immobilize Kv1.3 channels upon actin polymerization in HEK cells [26], and HS1 and Kv1.3 co-localize and interact in the IS upon IS formation in Jurkat T cells. In the IS, both WT channels and IL-2Rα were slightly slower than outside the IS—presumably due to some molecular interaction or crowding. Gaus et al. described a condensation of the T cell membrane on the site of T cell activation sustained by the actin cytoskeleton [44]. However, since the ΔC mutant in IS-engaged cells within the IS was not slower than outside the IS, it is probably not crowding but some specific interaction that causes the lower mobility. Previously, we reported enrichment of WT mGFP-Kv1.3, but not mGFP-Kv1.3-ΔC, in the IS. Enrichment was dependent not only on the C-terminal domain of the channel but also on the expression of PSD-95 [25]. The mobility measured at the IS cannot be explained by interactions via the C terminus either, as the mobility of the WT channel is higher, whereas that of the ΔC mutant is significantly lower in the IS than in standalone cells. Similar to standalone cells, external depolarization by high-K^+^ or MgTx reduced the mobility of WT and ΔC but not that of non-conducting mutant channels at the IS. Interestingly, in the IS, all proteins tested “became equal”; i.e., they had similar mobility regardless of size or mobility in standalone cells.

## 4. Materials and Methods

### 4.1. Cells and Cell Culture

We used Jurkat CD4^+^ T cell lines stably expressing different forms of Kv1.3 channels tagged with mGFP at the N-terminus: wild-type (WT), a ΔC mutant (the last 84 amino acids including the PDZ-binding domain deleted), and NON-CON mutant (point mutation in the poreregion (W384F), abolishing conductance but preserving motion of the gating charges). For control experiments, IL-2Rα was stably transfected into Jurkat cells expressing WT mGFP-Kv1.3 by retroviral transduction, as described in [32]. We used Raji B cells as antigen presenting cells that can form synapses with Jurkat cells. Jurkat and Raji cells were cultured in phenol-red free RPMI solution (Sigma-Aldrich, St. Louis, MO, USA) supplemented with 10% FBS, L-glutamine (2 mM), penicillin (100 U/L), and streptomycin (100 mg/L). Murine CH12.LX B cells and D10 T cells stably expressing GFP-PKCΘ and mCherry-Kv1.3 were cultured as described in [45]. Cells were maintained at 37 °C in a humidified, 5% CO_2_ atmosphere. Cells were passaged every 2–3 days.

### 4.2. Reagents, Fluorescence Labeling of Cells

We used Hanks’ Balanced Salt Solution (HBSS), referred to as “standard solution”, containing 5% glucose for washing the cells before the experiments. To attach the cells in 8-well microscopy chambers with coverslip bottoms (ibidi GmbH, Gräfelfing, Gemany or Nunc Lab-Tek, Thermo Fisher Scientific, Waltham, MA, USA), we coated the chambers with poly-L-lysine solution (1 mg/mL). To form immunological synapses, we used E type Staphylococcal Enterotoxin (SEE, 10 μg/mL, Toxin Technology Inc., Sarasota, FL, USA) as a superantigen. We depolarized cells either with a K-HBSS buffer, referred to as “high-K^+^ solution”, in which all Na^+^ ions were replaced by K^+^ (total [K^+^] = 150 mM; composition in mM: 1 CaCl_2_; 142.26 KCl; 0.44 KH_2_PO_4_; 0.75 MgSO_4_; 0.33 K_2_HPO_4_; 5.55 glucose; 10 HEPES; pH 7.4 adjusted with KOH), or by the Kv1.3 channel blocker margatoxin (MgTx, 1.5 nM, Alomone Labs, Jerusalem, Israel).

As controls in FCS experiments, we measured the mobility of MHC I molecules (targeted by Alexa 546-tagged W6/32 Fab, prepared from hybridoma [46]) and the DiIC_18_ fluorescent lipid analogue (Molecular Probes, Eugene, OR, USA) in Jurkat cells expressing the different mGFP-Kv1.3 variants. We chose DiIC_18_ because previously we found that changes of the membrane potential had no significant effect on its mobility [27]. As a further control, we measured the mobility of IL-2Rα labeled with Alexa-546-tagged anti-Tac Fab (Repligen Corporation, Needham Heights, MA, USA) in standalone and in IS-engaged Jurkat cells expressing WT mGFP-Kv1.3. Fab fragments were conjugated with Alexa 546 succinimidyl ester (Molecular Probes, Eugene, OR, USA). Before labeling with Fab-s, cells were washed twice with HBSS. They were then incubated with Fab-s (5 µg/10^6^ cells in 50 µL final volume) for 30 min on ice, washed twice in ice cold HBSS, and resuspended in HBSS. Before labeling with DiIC_18_, the stock solution of DiIC_18_ was sonicated, airfuged, and filtered through a 0.2 µm polycarbonate filter (Sigma-Aldrich, St. Louis, MO, USA) to get rid of microaggregates. We incubated the washed and resuspended cells with DiIC_18_ at a staining concentration of 1.5 µg/mL for 3 min at 37 °C. Cells were then washed and resuspended in HBSS [27].

### 4.3. Immunological Synapse (IS) Formation

Raji B cells (10^5^ cells) were incubated with 1 µg Staphylococcus enterotoxin E superantigen in 100 µL phenol-red free RPMI medium at 37 °C for 30 min. Cells were washed twice with 1 mL 37 °C phenol-red free RPMI. Cells were then suspended in 70 µL HBSS containing 5 mM glucose. In parallel, a Jurkat T cell suspension was made (10^5^ cells in 70 µL HBSS solution). Raji and Jurkat cells were mixed and spun in an Eppendorf centrifuge at 200× *g* for 1 min at 37 °C, then incubated for 10 min at 37 °C to form stable synapses. Cells were then resuspended gently to conserve synapses and seeded in microscopy chambers for FCS measurements.

### 4.4. Fluorescence Correlation Spectroscopy (FCS)

Molecular mobility was measured by fluorescence correlation spectroscopy. In FCS, a small (~0.25 μm^3^) selected volume element in the cell is illuminated by the focused laser beam of the confocal microscope, and the time course of the fluorescence intensity fluctuating due to molecular diffusion is detected. Measurements were performed on an Olympus FluoView 1000 confocal microscope (Olympus Europa Holding GmbH, Hamburg, Germany) equipped with a custom-made two-channel FCS unit attached to the 4th fluorescence port [47] or on a Zeiss LSM 880 confocal microscope having FCS capability. mGFP and Alexa 488 (used for calibration) were excited at 488 nm, while DiIC_18_ and Alexa 546 at 543 nm; 514/30 BP and 595 LP filters were used for detection, respectively. Autocorrelation functions (acf-s) were calculated by an ALV-5000 Multiple Tau Digital Correlator card attached to the Olympus (ALV, Langen, Germany) or by the built-in autocorrelator of the Zeiss microscope. Microscopes were equipped with incubator chambers, so FCS experiments were performed at 37 °C. In each standalone cell, 10 × 5 s runs were recorded at 3 selected points, whereas in IS-engaged cells, at 3 points inside the IS and 3 points outside the IS, elsewhere in the membrane. Measurements were carried out at the equator of the cell where membrane fluorescence appeared as a ring (Figure 3). This was necessary because the IS could not have been accessed otherwise.

For fitting autocorrelation functions, we used the QuickFit 3.0 program [48,49], applying a simulated annealing algorithm with box constraints, using standard deviations of the runs as weights. A model function with two freely diffusing species was used, the slow component accounting for the 2D diffusion of the channels in the cell membrane (along an axis perpendicular and another one parallel to the optical axis), and the fast component diffusing in 3D corresponding to channels having been internalized or being on route toward the membrane in vesicles. We also took into account triplet state formation and blinking [50]. The model is described by the following formula:

(1) G(τ)=1N⋅1−T−Θc+Te−ττtr+Θce−ττc1−T−Θc×[rfast(1+ττfast)−1(1+1S2ττfast)−1/2+rslow[(1+ττslow)(1+1S2ττslow)]−1/2]

where *N* is the average number of molecules in the detection volume, *τ* is the lag time, *τ_fast_* and *τ_slow_* are the diffusion times of the fast and slow diffusion components, *r_fast_* and *r_slow_* are the fractions of these components, and *S* is the axial ratio of the ellipsoidal detection volume. *T* denotes the equilibrium mole fraction of fluorophores in the triplet state [51,52] and *τ_tr_* is the triplet correlation time. Haupts et al. reported two independent protonation mechanisms of EGFP, an intramolecular proton transfer and a pH dependent external protonation process [50]. Since the characteristic time constants of the two protonation processes are separated by less than an order of magnitude at pH 7.4, a single term, characterized by the molecular fraction Θ_*c*_ and the correlation time *τ_c_*, was considered [53].

From the *τ_i_* diffusion times (*I* = fast or slow) of the examined molecule, the *D_i_* diffusion coefficients were calculated by comparing the diffusion parameters of the examined molecule to those of Alexa dye solutions. For mGFP-Kv1.3, Alexa 488; for DiIC_18_, Alexa546-tagged MHC I or IL-2Rα, Alexa 546 was used as a standard:

(2) $$D_{i}=\frac{\tau_{Alexa}\cdot D_{Alexa}}{\tau_{i}}$$

where *τ_i_* and *τ_Alexa_* is the measured diffusion time of the examined fluorescent protein and the used Alexa fluorescent dye. *D_Alexa488_* = 624 μm^2^/s and *D_Alexa546_* = 489 μm^2^/s are the estimated diffusion coefficients from the QuickFit 3.0 program [48] based on the known *D* values of the Alexa dyes (435 μm^2^/s for Alexa 488 and 341 μm^2^/s for Alexa 546) at 22.5 °C and extrapolating them to 37 °C, taking into account the change of water viscosity using the Stokes–Einstein relation [54]. The axial ratio *S* was also obtained from fitting the autocorrelation functions of the Alexa 488 solution, which was ~4.

### 4.5. Electrophysiology

Whole-cell patch-clamp experiments in current-clamp mode were performed to assess the membrane potential of Jurkat cell lines (WT and NON-CON mutant Kv1.3 expressing ones) with a Multiclamp 700B amplifier. The bath solution contained (in mM): 145 NaCl, 5 KCl, 1 MgCl_2_, 2.5 CaCl_2_, 5.5 glucose, 10 HEPES (pH 7.35). The pipette filling solution contained (in mM) 150 KCl, 2 MgCl_2_, 8.7 CaCl_2_, 5 HEPES, and 10 EGTA (pH 7.2). Cells to record were selected by using a Nikon TS100 fluorescence microscope.

### 4.6. Confocal Imaging

For imaging mGFP-Kv1.3 variants and F-actin, lone Jurkat cells or Jurkat cells forming an IS with Raji cells were used (5 × 10^5^ each). IS-s were formed as described above. After IS formation, they were centrifuged at 200× *g* for 1 min and incubated for 20 min at 37 °C, after which they were washed and fixed in 2% formaldehyde for 10 min. Cells were then permeabilized with 0.1% Tween 20 (Sigma-Aldrich, St. Louis, MO, USA). For F-actin labeling, cells were incubated with 0.1 µg/mL Alexa Fluor 546-Phalloidin (Thermo Fisher Scientific, Waltham, MA, USA) for 20 min. After multiple washing steps, Jurkat cells or Raji-Jurkat cell conjugates were resuspended in 2% formaldehyde and placed in 8-well chambered coverslips (ibidi GmbH). Images were acquired with a Nikon A1 (Nikon, Tokyo, Japan) confocal microscope equipped with a Plan-Apochromat 60×/NA 1.27 water immersion objective. Z-stacks consisting of 20–25 optical slices were collected by exciting the Alexa Fluor 546-phalloidin with a 561 nm laser and emission was captured by a PMT with a 593/46 nm filter. From the optical slices, we reconstructed the 3D distribution of F-actin using the NIS Elements software (Nikon) of the microscope.

Enrichment of Kv1.3 at the IS was calculated as described [32]. Briefly, the ratio of the average pixel intensity inside the IS and in the whole cell membrane was calculated from confocal images by using a MATLAB script as follows:

(3) $$\langle I_{IS,norm}\rangle =\frac{\sum I_{IS}/N_{IS}}{\sum I_{tot}/N_{tot}}$$

where *I_IS_* is the intensity of a pixel in the IS, *N_IS_* is the number of such pixels, *I_tot_* is the intensity of a pixel anywhere in the cell membrane (including the IS), and *N_tot_* is the number of all pixels.

### 4.7. Statistical Analysis

For the statistical analysis of the data, we used the Prism software (version 9.1.1. (225), GraphPad Software, San Diego, CA, USA). We applied analysis of variance (ordinary, one-way ANOVA) to determine statistical significance of differences between the means of investigated parameters. We made pairwise comparisons using unpaired *t*-test. Data are presented as box plots representing the 10th, 25th, 50th, and 90th percentiles and the arithmetic mean marked by a “+” sign. All slow diffusion coefficient values are summarized in Table 1.

## 5. Conclusions

Summing up our results, we can conclude that the C-terminus of Kv1.3 is critical in the observed low mobility of the channel in standalone cells, and based on our previous results, in getting enriched in the IS [25]. Besides the known role of PSD-95, interaction with the cytoskeleton may also be involved in lowering mobility of WT Kv1.3 in standalone cells [26], not observed for the ΔC mutant or IL-2Rα. Depolarization caused a decrease in the mobility of Kv1.3, similar to other membrane proteins [27]. Whereas mobilities were largely different in standalone cells, they became equal at the IS for all variants of Kv1.3 and even for IL-2Rα. At the IS, molecular crowding may reduce mobility relative to regions outside the IS.

## Figures and Tables

**Table 1:** Slow diffusion coefficient values of the examined molecules. Data are given in units of µm^2^/s as mean ± standard deviation. Values in the brackets denote the number of measured points. We used Jurkat T cells (standalone or forming an IS with Raji B cells) unless mentioned otherwise. * Diffusion constant of mCherry-Kv1.3 was also determined in murine D10 cells forming an IS with CH12-LX B cells, using a model with a single diffusing component.

		Diffusion Coefficient (µm^2^/s)								
		WT			Non-Conducting			ΔC		
		IS in	IS out	Standalone	IS in	IS out	Standalone	IS in	IS out	Standalone
Kv1.3	standard solution	**0.15** ± 0.069 (115)	**0.2** ± 0.11 (98)	**0.086** ± 0.061 (89)	**0.13** ± 0.062 (96)	**0.19** ± 0.079 (91)	**0.056** ± 0.037 (68)	**0.17** ± 0.095 (101)	**0.16** ± 0.073 (91)	**0.25** ± 0.11 (65)
	high K+ solution	**0.09** ± 0.05 (45)	**0.1** ± 0.055 (42)	**0.048** ± 0.022 (35)	**0.12** ± 0.062 (35)	**0.17** ± 0.06 (35)	**0.057** ± 0.037 (25)	**0.12** ± 0.04 (34)	**0.1** ± 0.03 (32)	**0.143** ± 0.063 (23)
	standard solution	**0.15** ± 0.01 (10)	**0.18** ± 0.09 (13)	**0.07** ± 0.02 (20)	**0.149** ± 0.1 (9)	**0.187** ± 0.09 (13)	**0.04** ± 0.028 (7)	**0.12** ± 0.045 (12)	**0.136** ± 0.016 (7)	**0.21** ± 0.066 (7)
	MgTx	**0.06** ± 0.03 (13)	**0.09** ± 0.055 (11)	**0.053** ± 0.021 (15)	**0.11** ± 0.08 (12)	**0.15** ± 0.06 (9)	**0.05** ± 0.012 (8)	**0.099** ± 0.03 (14)	**0.097** ± 0.03 (7)	**0.11** ± 0.04 (8)
	*in D10 cells* *	*0.021 ± 0.007 (15)*	*0.070 ± 0.007 (15)*							
MHC I	standard solution			**0.34** ± 0.23 (27)			**0.18** ± 0.13 (25)			**0.44** ± 0.31 (24)
DiIC_18_	standard solution			**0.54** ± 0.38 (19)			**0.68** ± 0.29 (25)			**0.72** ± 0.28 (28)
IL-2Rα	standard solution	**0.18** ± 0.09 (30)	**0.25** ± 0.137 (30)	**0.32** ± 0.087 (9)						

## Data Availability

All microscopy data are made available on request.

## Supplementary Materials

The supporting information can be downloaded at: https://www.mdpi.com/article/10.3390/ijms23063313/s1.
