# Supplementary material for: Role of C-Terminal Domain and Membrane Potential in the Mobility of Kv1.3 Channels in Immune Synapse Forming T Cells
Source: Int J Mol Sci. 2022 Mar 18;23(6):3313. doi: 10.3390/ijms23063313 (PMC8952507; doi:10.3390/ijms23063313)
Supplement: Supplementary file 1 [file ijms-23-03313-s001.zip › Sebestyen et al Supplementary material revised.pdf]

Supplementary material for

Role of C-terminal domain and membrane potential in the mobility of Kv1.3 channels in immune synapse forming T cells

Veronika Sebestyén, Éva Nagy, Gábor Mocsár, Julianna Volkó, Orsolya Szilágyi, György Panyi, Katalin Tóth, Péter Hajdu and György Vámosi

Supplemetary figures S1 – S7
Legends to Supplementary videos 1-8

A

mGFP-Kv1.3 WT

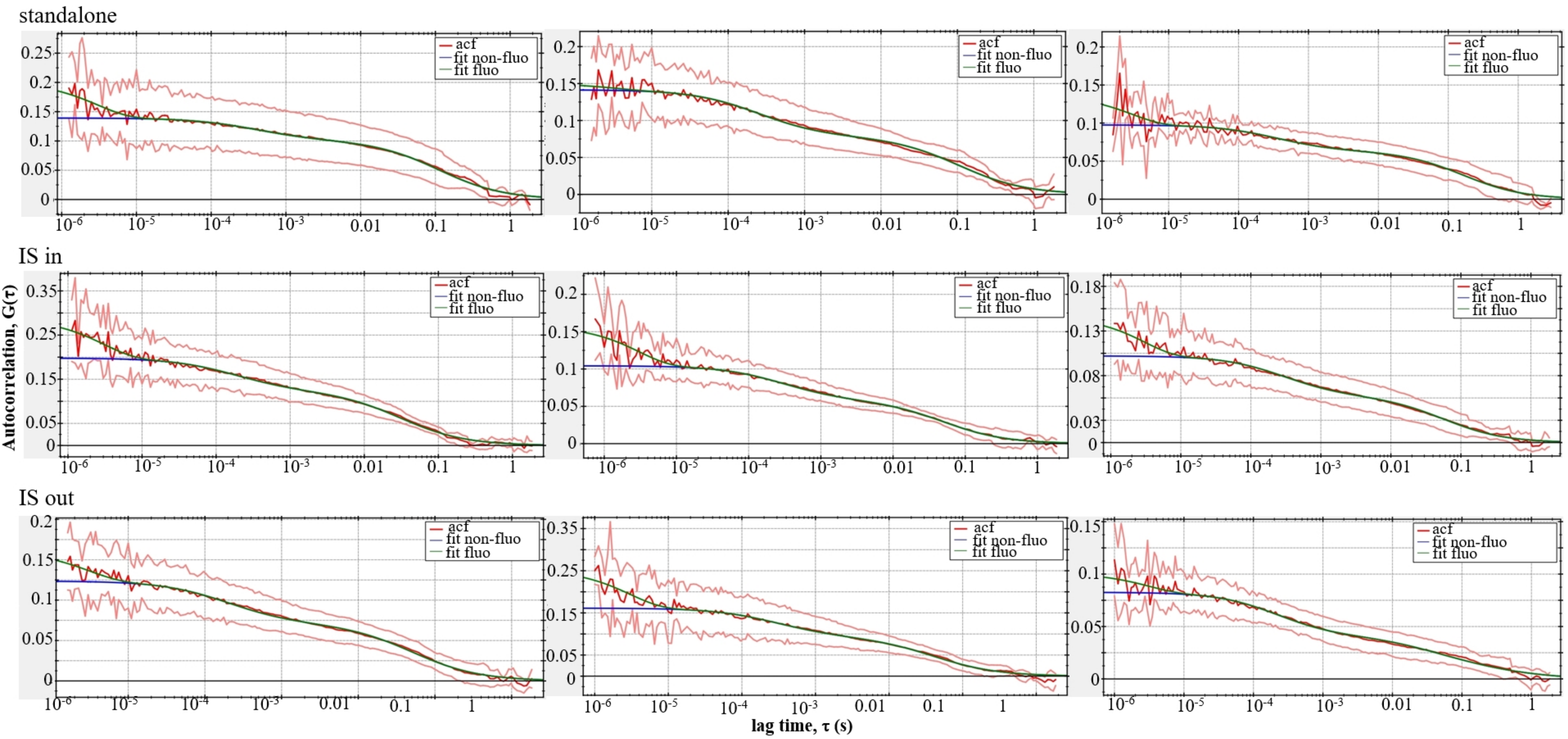

Fig. S1A: Autocorrelation curves of mGFP-Kv1.3 WT channels measured on standalone and synapse-forming Jurkat cells
Top: standalone cells; middle: in the IS; bottom: outside the IS (3-3 selected measurements). The dark red curves represent the mean autocorrelation funtions, the light-red curves above and below show standard deviations. The blue curve is the fit function neglecting the non-fluorescent components and the green one is the fit function considering them as well.

B

mGFP-Kv1.3 NON-CON

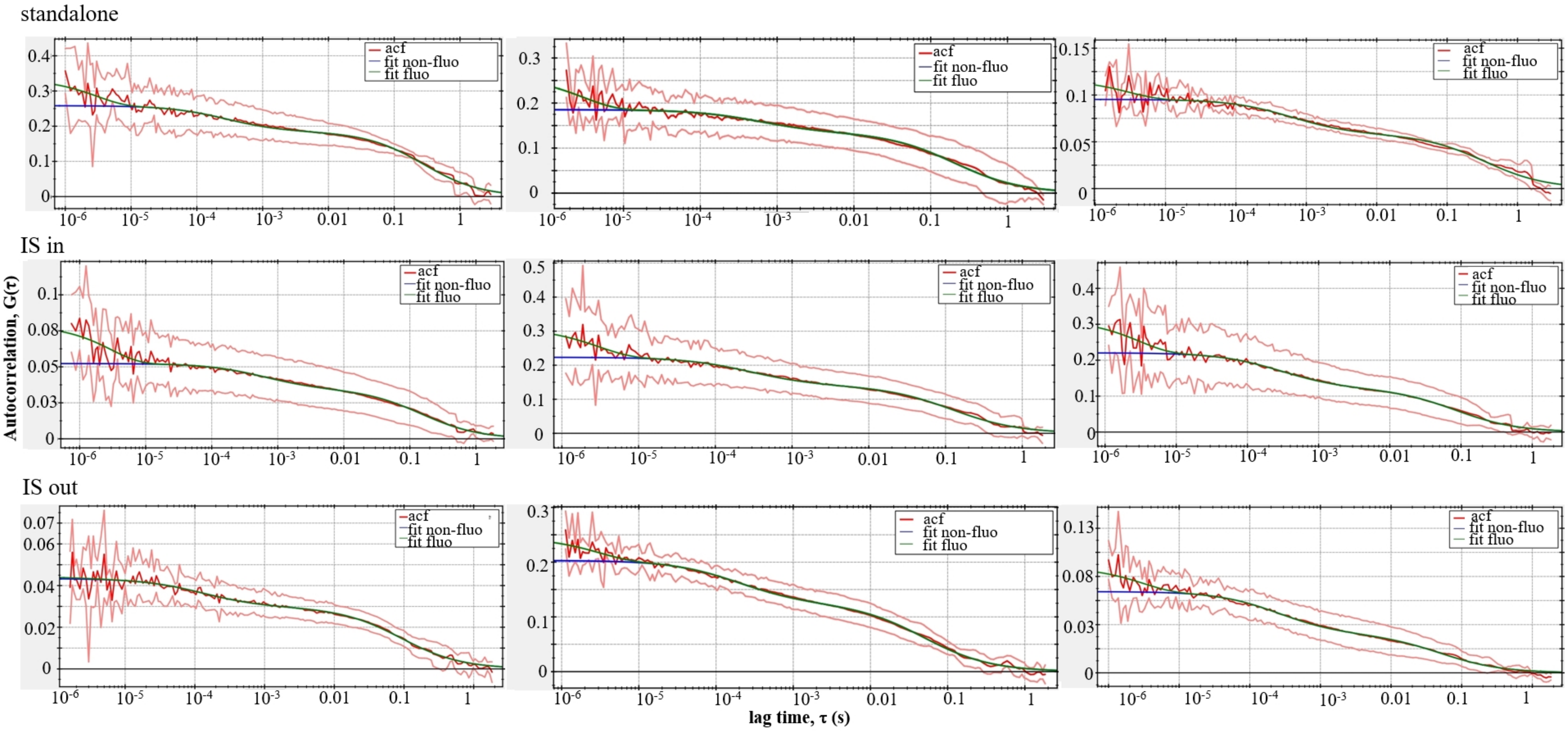

Fig. S1B: Autocorrelation curves of mGFP-Kv1.3 NON-CON channels measured on standalone and synapse-forming Jurkat cells
Top: standalone cells; middle: in the IS; bottom: outside the IS (3-3 selected measurements). The dark red curves represent the mean autocorrelation funtions, the light-red curves above and below show standard deviations. The blue curve is the fit function neglecting the non-fluorescent components and the green one is the fit function considering them as well.

C

mGFP-Kv1.3 ΔC

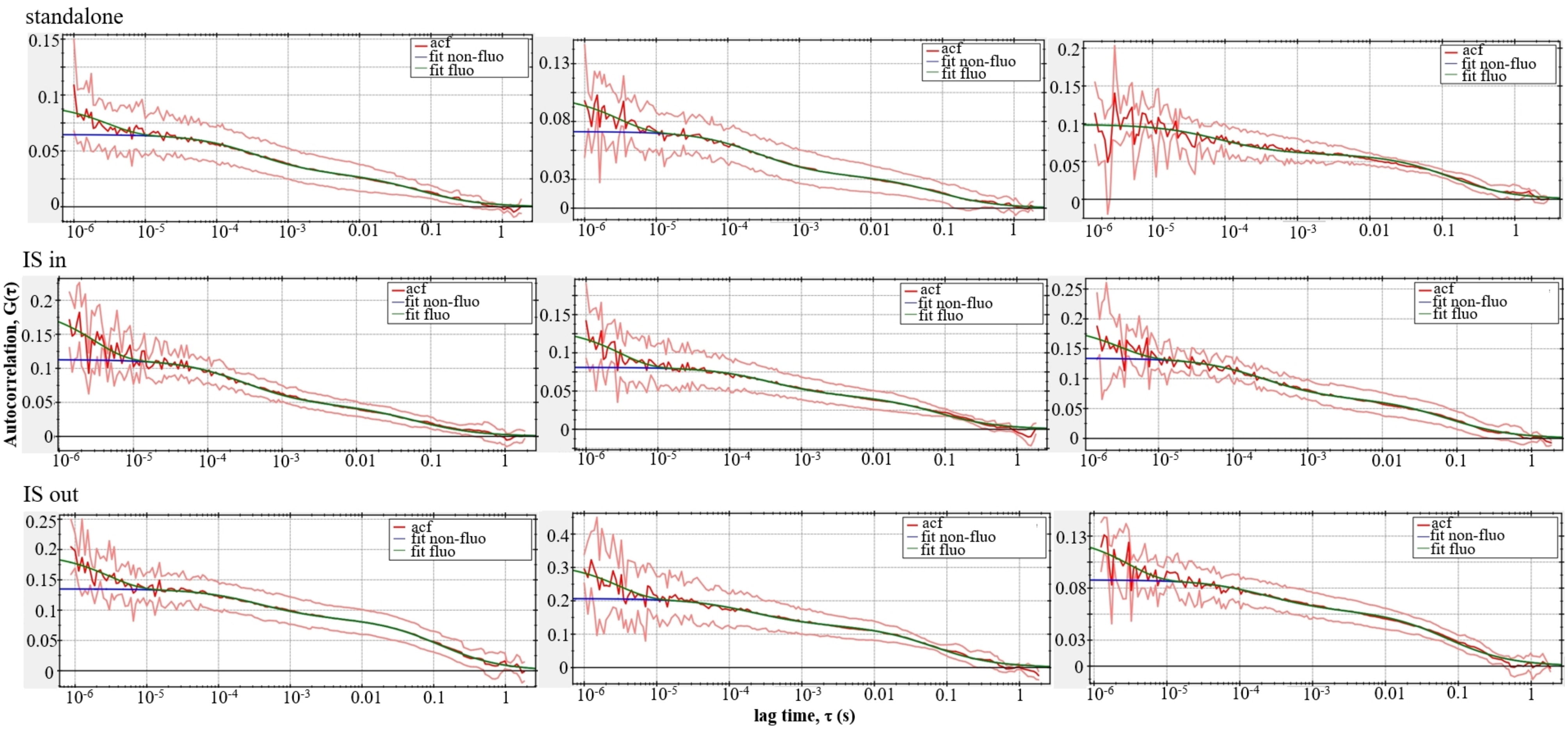

Fig. S1C: Autocorrelation curves of mGFP-Kv1.3 ΔC channels measured on standalone and synapse-forming Jurkat cells
Top: standalone cells; middle: in the IS; bottom: outside the IS (3-3 selected measurements). The dark red curves represent the mean autocorrelation funtions, the light-red curves above and below show standard deviations. The blue curve is the fit function neglecting the non-fluorescent components and the green one is the fit function considering them as well.

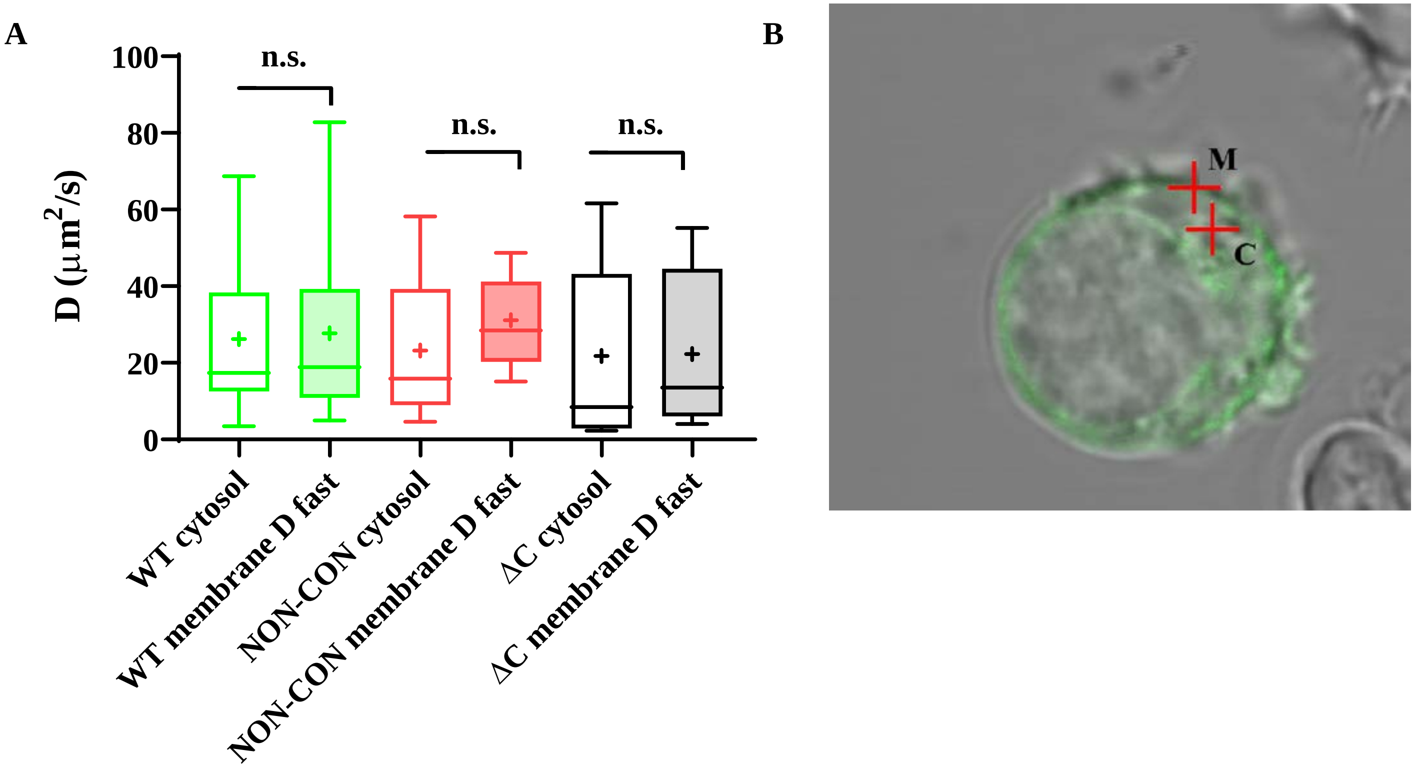

Fig. S2: Mobility of mGFP-Kv1.3 variants in the cytosolic region and in the membrane of standalone cells
(A) Diffusion constants of mGFP-Kv1.3 variants measured in the cytosol (open boxes) and fast component measured in the cell membrane (filled boxes) of standalone Jurkat cells. Autocorrelation curves for cytosolic regions could be fit with a assuming a single component diffusing in 3D. Curves from the membranes were fit with two diffusing components, from which the fast component is shown. (B) Transmission and fluorescence microscopic image of an mGFP-Kv1.3 WT channel expressed in a Jurkat cell. The cross marked with M represents a typical position where “in-membrane” measurements were performed, while C represents a location of cytosolic measurements.

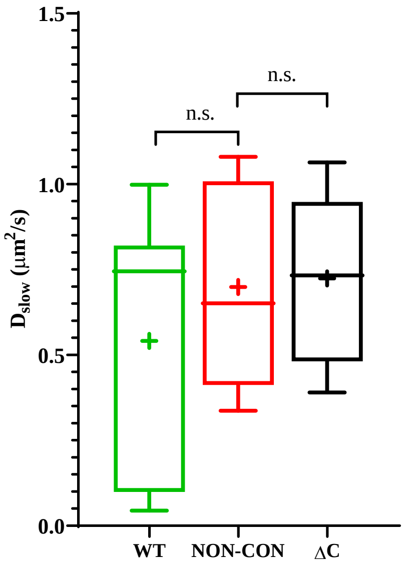

Figure S3: Mobility of $DiIC_{18}$ fluorescent lipid analogue on standalone Jurkat cells expressing WT, non-conducting or ΔC mutant mGFP-Kv1.3 channels. n.s., not significant.

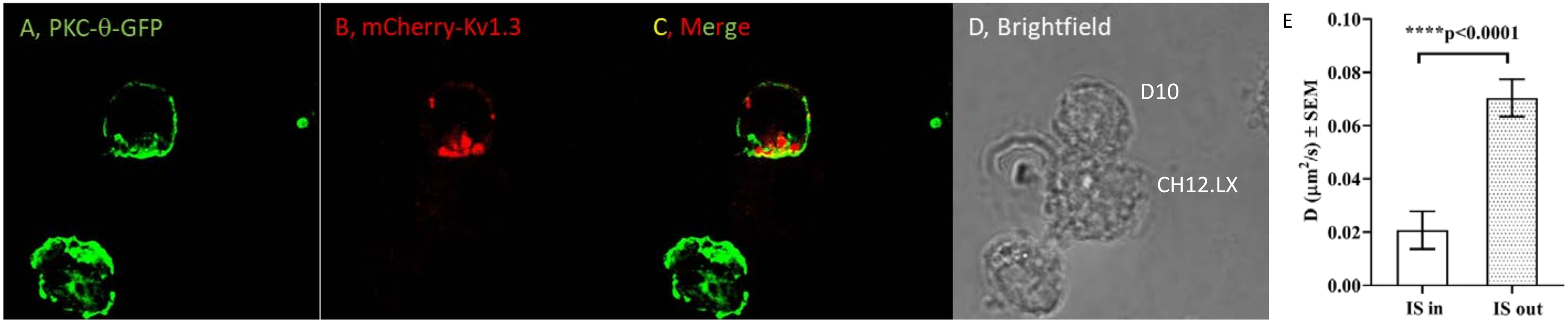

Figure S4: Mobility of mCherry-Kv1.3 on D10 T cells. We also investigated Kv1.3 in a murine model system: the conalbumin-pulsed CH12.LX B cell line and the D10 T (Th2) cell line stably expressing GFP-PKCΘ and mCherry-Kv1.3. D10 cells recognize conalbumin antigen presented by the CH12-LX B cells. Prior to the measurements, CH12-LX cells were incubated overnight with 500 µg/ml conalbumin from chicken egg white in phenol-red free medium. T and B cells were then mixed at a 1:1 ratio. The mixture of cells was incubated at 37 °C for 15-30 min to form IS-s. After IS formation, GFP-PKCΘ (panel A) and mCherry-Kv1.3 (panel B) translocated to the IS (images were taken 15 min after conjugation). C) Merge of green and red channels. D) Brightfield image. E) Mobility of mCherry Kv1.3. in the D10 murine T cells. Autocorrelation curves were fitted with a single diffusion component. We could detect a significant decrease in the mobility of Kv1.3 channels within the IS compared to non-synaptic membrane regions (****$p<0.0001$).

Standalone Jurkat cell

IS-engaged Jurkat cell

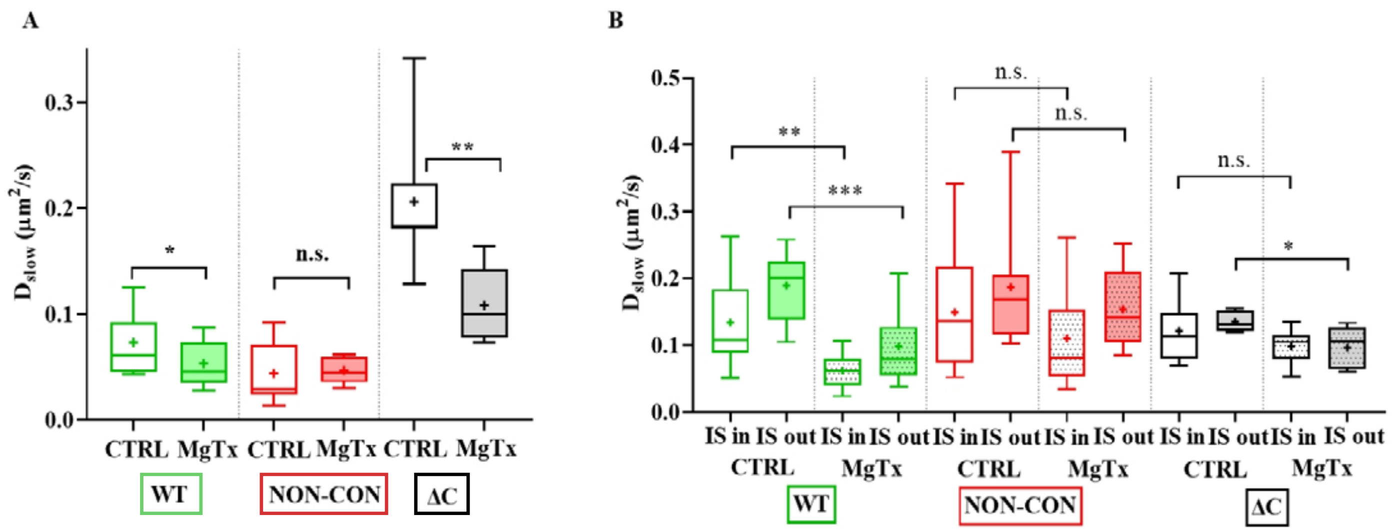

Figure S5: Effect of depolarization with margatoxin on the slow component of the Kv1.3 channel's mobility A) on standalone and B) on synapse forming cells.

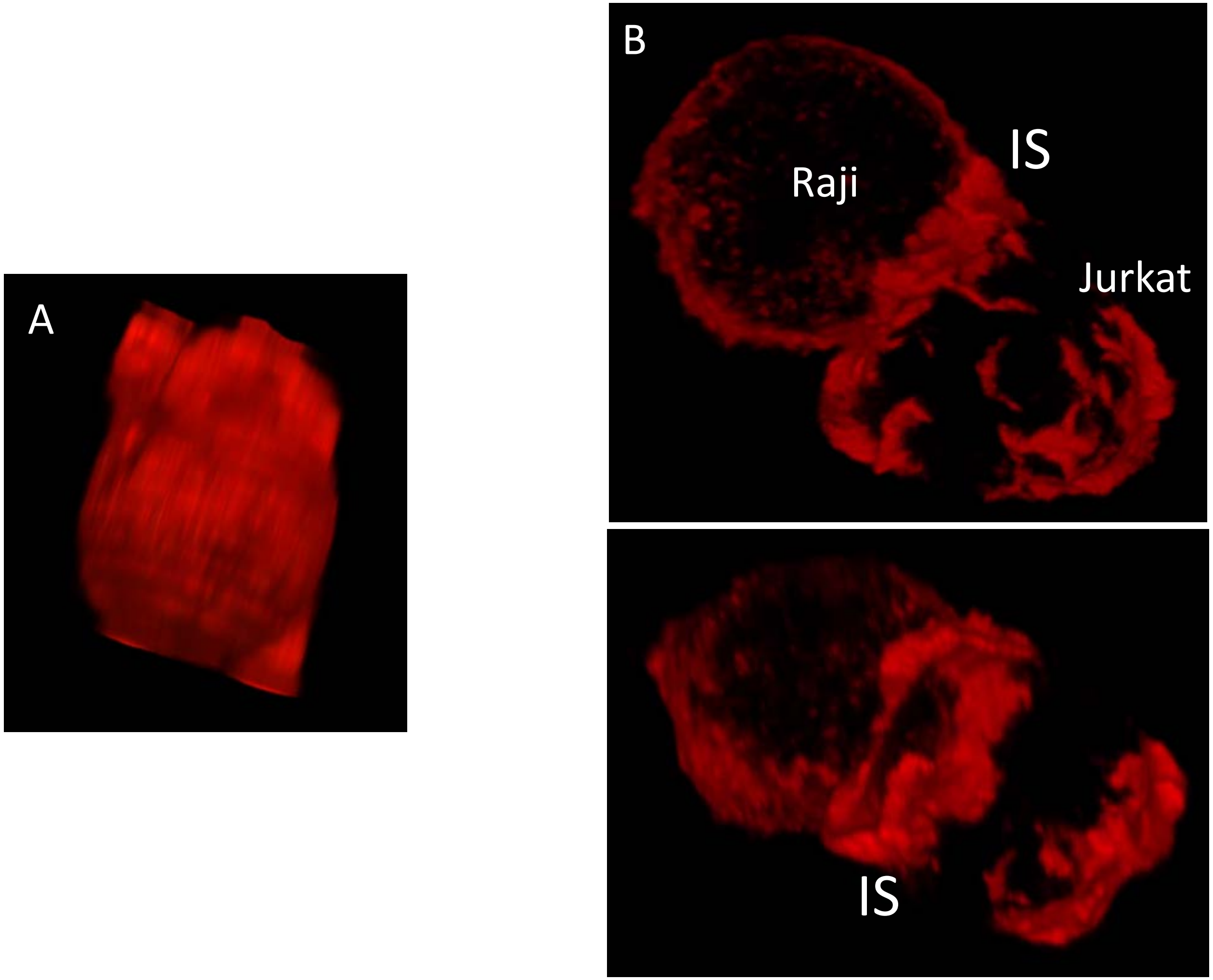

Figure S6: 3D reconstruction of the actin cytoskeleton labeled with Alexa 546-phalloidin. 3D reconstruction was created from the optical slices recorded with a Nikon A1 confocal microscope using the NIS Elements software. Images shown are snapshots from **Supplementary videos 1 and 2.**

A) Standalone Jurkat cell. Cortical F-actin is present in most areas of the cell membrane. **B)** Two different views of F-actin in Jurkat cell forming an IS with a Raji cell. In the Jurkat cell F-actin forms a ring around the contact area at the IS (bottom panel), but the central area inside the IS is hardly stained. In the rest of the Jurkat cell, F-actin is polarized to the side opposite to the IS, leaving some of the area in between void of F-actin.

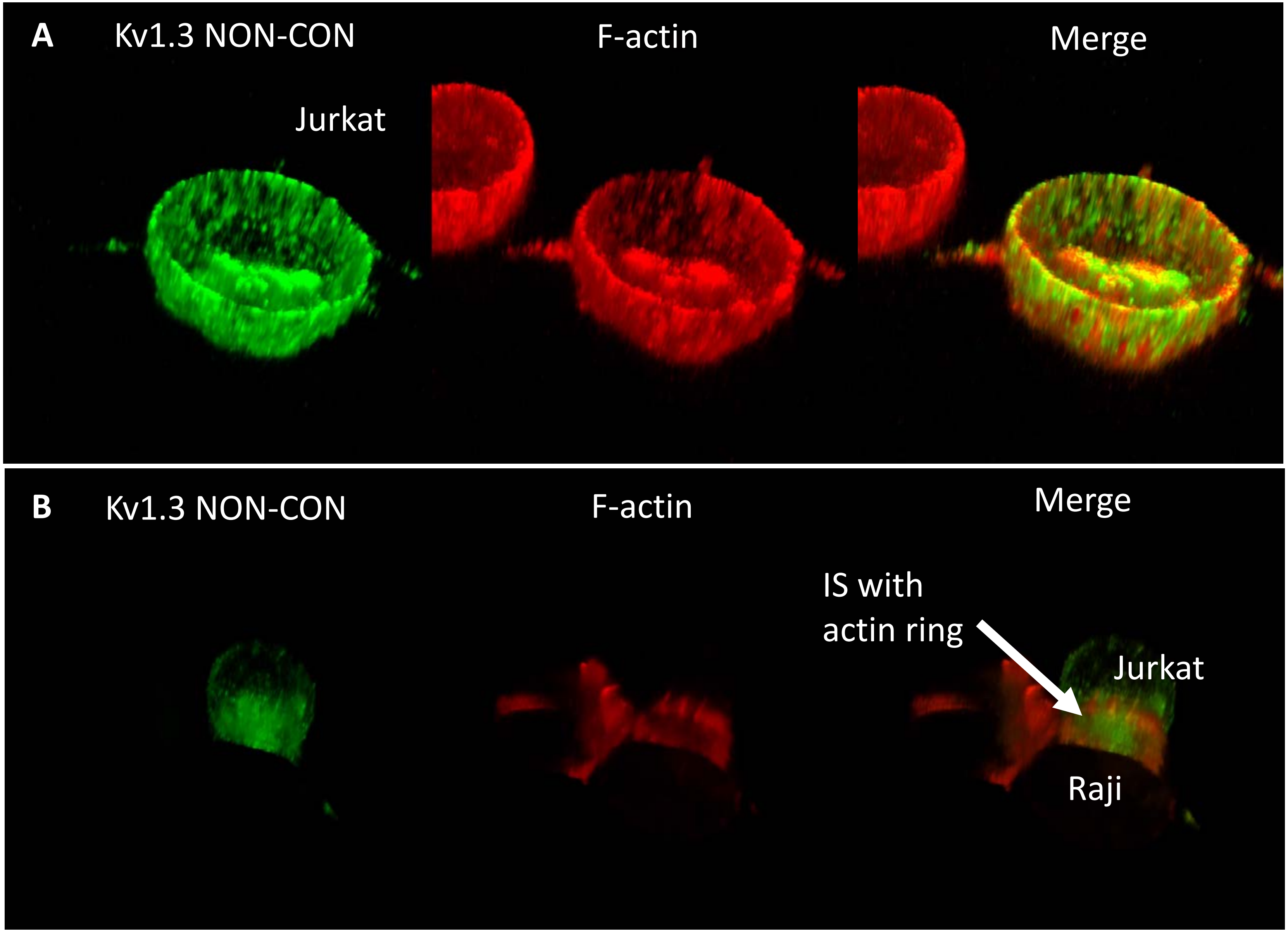

Fig. S7: Distribution of NON-CON and ΔC mutants of Kv1.3 and F-actin in standalone and IS-engaged Jurkat cells
(legend see on next page)

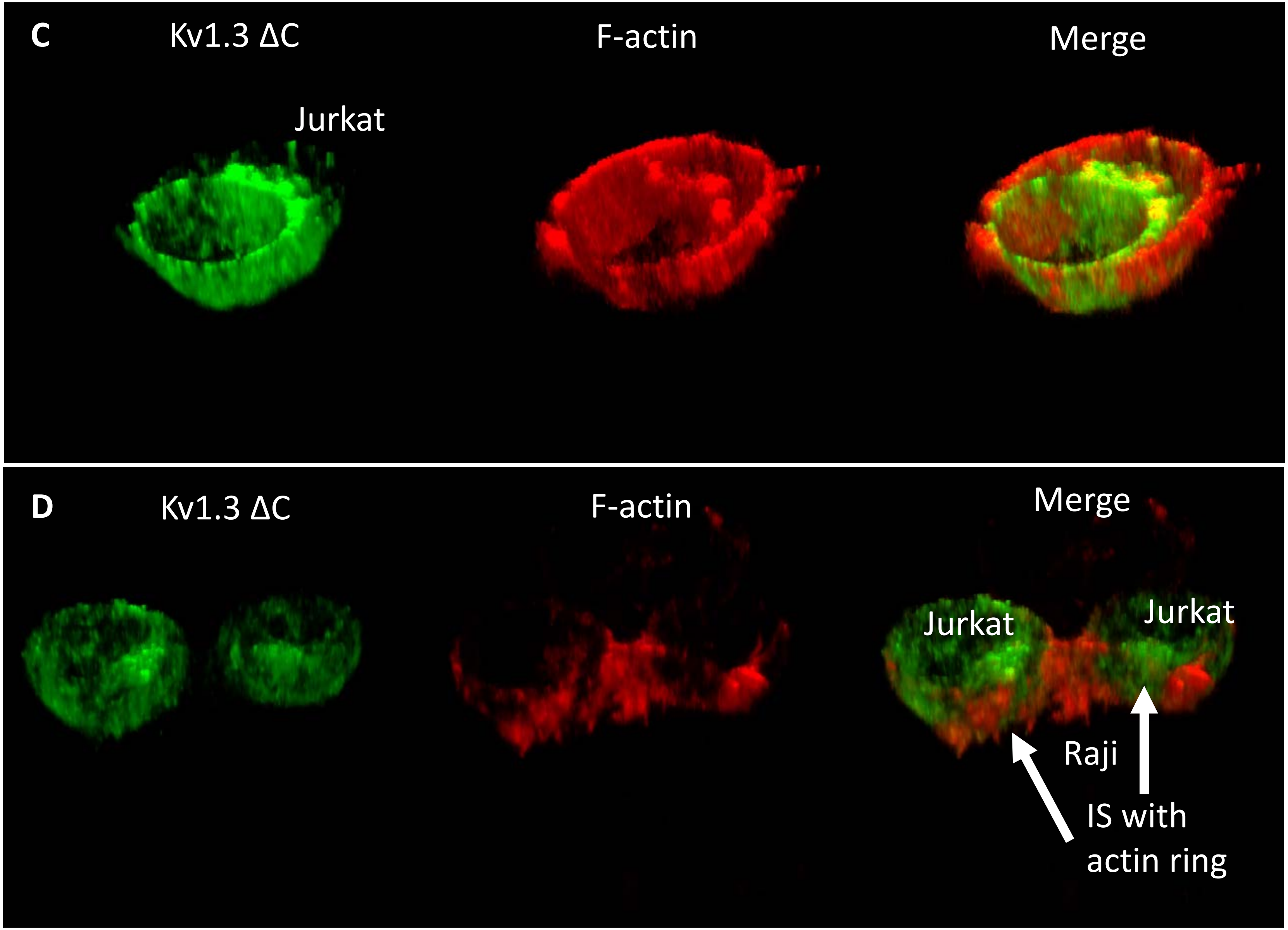

Fig. S7: Distribution of NON-CON and ΔC mutants of Kv1.3 and F-actin in standalone and IS-engaged Jurkat cells
Kv1.3 was tagged with mGFP and F-actin was labeled with Alexa546-phalloidin. 3D reconstructions from z-stacks of confocal images are shown. A) Standalone Jurkat cell with Kv1.3 NON-CON, B) Jurkat cell with Kv1.3 NON-CON in IS with Raji cell. C) Kv1.3 ΔC, D) Kv1.3 ΔC in IS. The arrows mark the IS and the acting ring formed at its periphery.

Supplementary videos 1 and 2: 3D reconstruction of F-actin labeled with Alexa 546-phalloidin. 3D reconstruction was created from the optical slices recorded with a Nikon A1 confocal microscope using the NIS Elements software. **Figures S6A,B** are snapshots from these videos.

video 1: Standalone Jurkat cell. Cortical F-actin is present in most areas of the cell membrane.

video 2: F-actin in Jurkat cell forming an IS with a Raji cell. In the Jurkat cell F-actin forms a ring at the periphery of the IS, whereas the central area inside the IS is hardly stained. In the rest of the Jurkat cell, F-actin is polarized to the side opposite to the IS, leaving the area almost void of F-actin between the opposite pole and the IS.

Supplementary videos 3-8: 3D reconstruction of the distribution of mGFP-Kv1.3 variants and F-actin labeled with Alexa 546-phalloidin in standalone Jurkat cells and in Jurkat cells forming an IS with Raji cells. F-actin is labeled in both cell types. 3D reconstruction was created from the optical slices recorded with a Nikon A1 confocal microscope using the NIS Elements software. **Fig. 6 and Fig. S7** are snapshots from these videos.

video 3: Standalone Jurkat cell expressing mGFP-Kv1.3 WT.

video 4: Jurkat cell expressing mGFP-Kv1.3 WT forming an IS with Raji cell.

video 5: Standalone Jurkat cell expressing mGFP-Kv1.3 NON-CON mutant.

video 6: Jurkat cell expressing mGFP-Kv1.3 NON-CON mutant forming an IS with Raji cell.

video 7: Standalone Jurkat cell expressing mGFP-Kv1.3 ΔC mutant.

video 8: Jurkat cell expressing mGFP-Kv1.3 ΔC mutant forming an IS with Raji cell.
